# Pharmacological Inhibition of Spleen Tyrosine Kinase Suppressed Neuroinflammation and Cognitive Dysfunction in LPS-Induced Neurodegeneration Model

**DOI:** 10.3390/cells11111777

**Published:** 2022-05-28

**Authors:** Min Woo Kim, Kyonghwan Choe, Jun Sung Park, Hyeon Jin Lee, Min Hwa Kang, Riaz Ahmad, Myeong Ok Kim

**Affiliations:** 1 Division of Life Sciences and Applied Life Science (BK 21 Four), College of Natural Science, Gyeongsang National University, Jinju 52828, Korea; mwkim0322@gnu.ac.kr (M.W.K.); k.choe@maastrichtuniversity.nl (K.C.); jsp@gnu.ac.kr (J.S.P.); lhj4912@gnu.ac.kr (H.J.L.); kmh1020@gnu.ac.kr (M.H.K.); riazk0499@gnu.ac.kr (R.A.); 2 Department of Psychiatry and Neuropsychology, School for Mental Health and Neuroscience (MHeNs), Maastricht University, 6229ER Maastricht, The Netherlands; 3 Alz-Dementia Korea Co., Jinju 52828, Korea

**Keywords:** lipopolysaccharide, neuroinflammation, neurodegeneration, microglia, Syk, cognitive and memory dysfunctions

## Abstract

Tyrosine-protein kinase (Syk) plays a potential role in neuroinflammation and adaptive immune responses in several neurodegenerative conditions. Seeing the significant role of Syk in the pathophysiology of neurodegeneration, several pharmacological inhibitors have been developed. One of the known inhibitors of Syk is BAY61-3606, which has shown efficacies in Alzheimer’s disease (AD) through regulating amyloid production. However, little is known about its efficacies in neuroinflammation and neurodegeneration. Our finding showed that Syk expression was up-regulated by lipopolysaccharide (LPS)-dependent manner, and BAY61-3606 significantly suppressed the activated microglia (ionized calcium-binding adaptor molecule 1 [Iba-1]) and the inflammatory cytokines (tumor necrosis factor-alpha [TNF-α], interleukin 1-beta [IL-1β], IL-6) and other inflammatory mediators (nuclear factor kappa B [NF-κB], cyclooxygenase-2 [Cox-2], and inducible nitric axide synthase [iNOS]) in the lipopolysaccharide (LPS)-treated in vivo and in vitro models. Moreover, BAY61-3606 significantly reduced microglia-mediated neuronal cell death by regulating the expression of Cytochrome C and Bim (B-cell lymphoma 2 [BCL-2] interacting mediator of cell death) in the LPS-treated mice brain and HT22 cells. Furthermore, the expression of synaptic markers, synaptosomal-associated protein, 25 kDa (SNAP25), synaptophysin (Syp), and postsynaptic density protein-95 (PSD95) in LPS-challenged mice showed that BAY61-3606 significantly recovered the synaptic markers. Finally, we have analyzed the effects of BAY61-3606 against memory and cognitive dysfunctions in the LPS injected mice. The Y-maze test and Passive avoidance test suggested that BAY61-3606 significantly protected against LPS-induced cognitive and memory dysfunctions. The current findings not only highlight the mechanisms of Syk in the pathophysiology of neuro-inflammation, but also support the therapeutic efficacy of BAY61-3606 in the management of neurodegeneration.

## 1. Introduction

Neuroinflammation is initiated from aging and various pathological conditions such as neurodegenerative diseases, including Alzheimer’s disease (AD) and Parkinson’s disease (PD), and systemic inflammation induced by metabolic dysfunction [1,2,3]. Microglia is the major cell which regulates inflammation in the brain, which is a lineage of macrophages. One of the many functions of microglia is to observe the microenvironment encompassing them and maintain brain homeostasis; scavenging debris from damaged or unnecessary neurons, phagocytosis, antigen presentation, and synaptic pruning [4]. Although microglia activation serves to maintain brain homeostasis, it mediates neurotoxicity when it becomes overactive by external stress, including aging or pathological conditions [5]. Neurotoxicity is influenced by pro-inflammatory cytokines and inflammatory mediators secreted from microglia, finally causing neuronal cell death and neuronal dysfunction [6]. Therefore, blocking microglia activation is a primary target to regulate neuroinflammation induced by pathological conditions.

Lipopolysaccharides (LPS), the outer membrane of gram-negative bacteria, is a ligand of Toll-like receptor 4 (TLR4) widely used to initiate inflammatory responses, including central nervous system (CNS) [7]. LPS-primed microglia mediate inflammatory action through activating and secreting a variety of pro-inflammatory cytokine and mediators, such as tumor necrosis factor alpha (TNFα), interleukin 1-beta (IL)-1β, cyclooxygenase-2 (COX-2), and inducible nitric oxide synthase (iNOS), followed by nitric oxide (NO) secretion [8]. Additionally, these activating microglia can promote neuronal loss and neurodegeneration as well as other glial cell activation. Previous in vivo study reported that mice treated with LPS exhibited body weight loss, cognitive dysfunction, and depressive behavior including anxiety, decreased locomotion, and exploration, which encompasses features of neurodegenerative diseases [9]. Therefore, the LPS model is considered the most effective way to study microglia-mediated neuroinflammation and neurodegeneration.

Tyrosin-protein kinase (Syk) is a crucial regulator of various signaling transduction. Syk is involved in adaptive immunity and phagocytic activities of the macrophages. Studies have shown that Syk-mediated nuclear factor kappa B (NF-κB) activation is associated with IL-1β and TNFα. Additionally, microglial Syk responds to external stress, including amyloid-beta (Aβ) oligomer, and ischemic stroke [10,11,12]. Upon activation at the microglial level, the Syk causes the production of reactive oxidative and nitrosative stress [13], which is responsible for the disruption of cell membranes and cellular DNA damage [14]. 

Inversely, it has also been reported that inhibition of Syk has shown efficacies in Aβ reduction and ischemic stroke [15,16,17]. Therefore, multiple studies are trying to suppress Syk as a therapeutic way of pathological status. BAY61-3606, a selective Syk inhibitor, has been widely utilized to suppress various inflammation-mediated pathological models including sepsis, acute kidney injury, and traumatic brain injury through modulating macrophage activation [18,19,20]. In the brain, it has been reported that Syk implicates to brain tissue damage from ischemia and AD proteopathy. In particular, the decrease in Aβ and hyperphosphorylated tau are likely to be associated with the increase in microglia phagocytosis following Syk inhibition [17]. Conversely, Syk is highly activated in AD mouse model like transgenic (Tg) APPsw and Tg PS1/APPsw, and overexpression of Syk promotes total tau and hyperphosphorylated tau in SH-SY5Y cells [21]. Additionally, BAY61-3606 completely inhibits Tau phosphorylation at Ser-396/Ser-404 (PHF-1), which implicates synaptic dysfunction and cognitive impairment in human neuroblastoma SH-SY5Y cells [22]. Due to its pleiotropic actions in neuropathological conditions, identifying the role of brain Syk has been of great importance in current research. Although significance of Syk has been gradually highlighted in CNS, association of neuroinflammation mediated by microglial Syk and cognitive decline and neurodegeneration has not been well understood.

**Figure 1:**
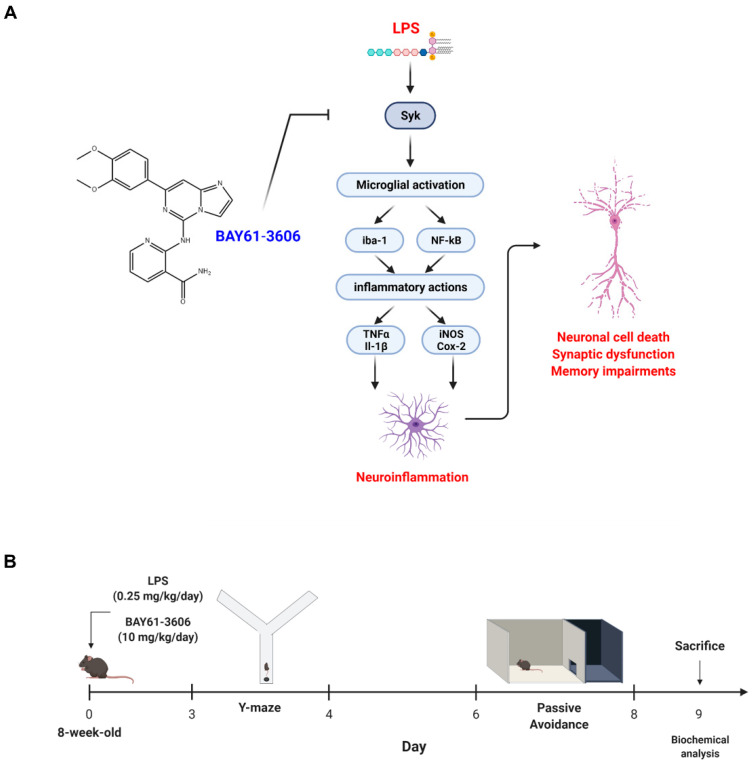
Study Design and Graphical abstract for the current study. (**A**) LPS stimulates microglial cells via enhancing the expression of Syk. Sequentially, microglia execute inflammatory actions through activation of NF-κB, inflammatory mediators, and pro-inflammatory cytokines. This neurotoxin promotes synaptic dysfunction and neuronal cell death, and finally leads to memory impairments. Pharmacological inhibition of Syk (via BAY61-3606) suppressed the overall inflammation and neurodegeneration in LPS-treated in vivo and in vitro models. (**B**) Experimental scheme of LPS-induced mice model. LPS or LPS+BAY61-3606 were intraperitoneally treated in 8-week-old male mice until termination of experiment. Two kinds of behavior tests, Y-maze (3 days) and passive avoidance (6 days), were performed to measure spatial working memory and learning memory. Biochemical analyses were proceeded at the end of the behavior test. Abbreviations: LPS, lipopolysaccharide; Syk, tyrosine-protein kinase; NF-κB, nuclear factor kappa B.

Therefore, in this study, we investigated whether pharmacological inhibition of Syk, through BAY61-3606, suppress LPS-induced neuroinflammation, cognitive dysfunction, and neurodegeneration. Our results indicated that Syk inhibition reduced LPS-induced microglia activation and thereby promoted neuronal survival and recovered cognitive dysfunction. These findings suggest that Syk inhibition may be a promising therapeutic target for neuroinflammation-mediated neurodegeneration (Figure 1A).

## 2. Materials and Methods

### 2.1. Animal

Seven week-old male mice (*n* = 4 per group) (C57BL/6 background) were purchased from Samtako Bio (Osan, Korea). On the first week, mice were housed in a facility providing automatic 12 h light/12 h dark cycle, humidity (60 ± 15%), and temperature (23 ± 2 °C) for assimilation. Mice were fed ad libitum food and water. The groups were divided as shown below, and all chemicals (LPS (L2630, Sigma Aldrich, St.Louis, MO, USA), BAY61-3606 (11423, Cayman, Ann Arbor, MI, USA)) were treated by intraperitoneal injection. Figure 1B describes the overall schematic of the experimental schedules.

1. Control group (Saline for 9 days).
2. LPS group (0.25 mg/kg for 9 days).
3. LPS + BAY61-3606 (0.25 mg/kg + 10 mg/kg for 9 days).
4. BAY61-3606 (10 mg/kg for 9 days).

### 2.2. Y-Maze and Passive Avoidance Task

For Y-maze task, mice were placed at the end of one arm and allowed to freely move around the maze for 8 minutes (min). After every experiment, the maze was sterilized with 70% ethanol to avoid direction bias. The total arm entries, spontaneous alternation, and mean zone speed were recorded by SMART V3.0 (Harvard apparatus, Holliston, MA, USA).

For the passive avoidance task, mice explored freely in the light chamber with the condition of a closed dark chamber for 3 min on the first day. On day 2, mice were placed in the lightbox. After 30 s, the dark chamber door was opened. When mice passed through the dark chamber, the door was closed and gave an electric shock derived from the grid floor (0.2 mA, 2 seconds [s]). After 24 hours (h) of stabilization, mice were again placed in the lightbox with the same conditions as day 2 except for giving an electric shock, and the entry latency was measured when they passed through the dark chamber with a cutoff time of 5 min. 

### 2.3. Tissue Processing

Tissue processing was performed as previously reported with some modifications [23,24,25]. Mice were transcardially perfused with ice-cold phosphate-buffered saline (PBS) and 4% paraformaldehyde and sequentially postfixed for 48 h in 4% paraformaldehyde and 20% sucrose for 24 h. Brains were embedded in the matrix containing optimal cutting temperature (O.C.T.) compound and frozen in liquid nitrogen. Finally, brain slices were sectioned with 20 μm thickness using cryostat CM 1950 (Leica, Deer Park, IL, USA). Tissue-containing slides were stored at −70 °C and incubated overnight at room temperature at every use before staining and immunofluorescence. Staining and immunofluorescence were observed at caudal diencephalon of mice brain.

### 2.4. Nissl Staining

Nissl staining was performed as previously reported with some modifications [26]. After brain slices were dehydrated with xylene and rehydrated in 100% EtOH, the slides were incubated with 0.1% cresyl violet for 15 min, washed in 70% ethanol, and immersed with differentiation solution. After being cleared in xylene for 3 min, the slides were mounted with dibutylphthalate polystyrene xylene (D.P.X) mounting medium under a coverslip. Nissl-positive cells were observed by light microscopy (Axioskpo2 plus, Zen, Zeiss, Oberkochen, Germany) and analyzed by ImageJ software (Ver.1.53o, National Institutes of Health, Bethesda, MD, USA).

### 2.5. Immunofluorescence 

Immunofluorescence was performed as previously reported with some modifications [27]. Citrate buffer-based heat-induced antigen retrieval was performed with brain hippocampal slices at 9–100 °C for 20 min. After 10 min of drying, slides were rinsed in PBS, including 0.1% Tween 20 (PBST), and immersed with blocking solution containing 5% normal goat serum and 0.1% TritonX-100 in PBST for 1 h at room temperature (RT). Then, the sections were incubated with primary antibodies diluted in blocking solution at 4 °C in a humidified chamber. The next day, the slides were exposed to room temperature for 1 h, washed in PBST, and incubated with a secondary antibody, goat-anti mouse conjugated with Alexa fluor 488 or rabbit conjugated with Alexa fluor 594 (Thermo Fisher scientific, Waltham, MA, USA) for 90 min at RT. After washing the slides, 4′,6′-diamidino-2-phenylindole (DAPI) was used for 10 min, and sections were mounted under the coverslips. The target protein of interest was detected by confocal laser-scanning microscopy (FV 1000MPE, Fluoview, Olympus, Tokyo, Japan).

### 2.6. Cell Culture

Microglia cell line BV2 were maintained in Dulbecco’s modified Eagle’s medium (DMEM) (Gibco, Waltham, MA, USA) supplemented with 5% fetal bovine serum (FBS) (Gibco, Waltham, MA, USA) and 1% penicillin/streptomycin (Thermo Fisher Scientific, Waltham, MA, USA). Mouse hippocampal cell line HT22 was maintained in DMEM, supplemented with 10% FBS and 1% penicillin/streptomycin (Gibco, Waltham, MA, USA). LPS (Sigma Aldrich, St.Louis, MO, USA) was dissolved in 1X PBS with a stock concentration of 1 mg/mL. BV2 was seeded with 2.0 × 10^5^ cells/mL. When the cell confluency reached 70%, 100 ng/mL of LPS was introduced for 6 h to isolate RNA and 24 h to isolate protein. For detecting LPS-induced nitrite from microglia media, Griess reagent was used according to the manufacturer’s instructions. After treating LPS-primed conditioned media in HT22 for 24 h, cell viability was measured using 3-(4,5-Dimethylthiazol-2-yl)-2,5-Diphenyltetrazolium Bromide (MTT). 

### 2.7. Transwell Co-Culture Assay

Microglial BV2 cells were plated on transwell (0.4 μm pore) and mouse hippocampal HT22 were plated in 6-well plates (Corning Incorporated, Corning, NY, USA). BV2 were treated with LPS (100 ng/mL) and with or without BAY61-3606 (10 nM and 100 nM) for 24 h. At the endpoint of the experiment, media from transwell and 6-well plates were collected to measure nitrite and HT22 were collected for western blotting to assess neuronal cell death.

### 2.8. Western Blot Analysis

Tissue and cells were lysed in radio-immunoprecipitation assay (RIPA) buffer (GeneDEPOT, Katy, TX, USA) containing phosphatase and protease inhibitor cocktail (GeneDEPOT, Katy, TX, USA). The total lysate was centrifuged at 13,000 rpm at 4 °C for 30 min. Protein concentration was quantified by the Bradford assay (BioRad, Hercules, CA, USA). 30 μg of protein was resolved by SDS-PAGE and transferred to the poly(vinylidene fluoride) (PVDF) membrane (Whatman, Kent, ME, USA). Primary antibodies with the desired dilution were treated on a transferred membrane at 4 °C overnight followed by horseradish peroxidase (HRP)-conjugated secondary antibodies. Signals were detected by Fuji medical X-ray film (Fuji, Chiryu, Japan). Optical densities were normalized with β-actin using Image J software (Ver.1.53o, National Institutes of Health, Bethesda, MD, USA).

### 2.9. Antibodies

BCL-2-like protein 11 (Bim) (#2933) at 1:3000, Syk (#13198) at 1:3000, Caspase 3 (#9662) at 1:100 (Cell Signaling Technology, Danvers, MA, USA), iba-1 (016-20001) at 1:1000 (WAKO, Tokyo, Japan), β-actin (sc-47778) at 1:10,000, Cytochrome C (sc-13156) at 1:3000, Glial fibrillary acidic protein (GFAP) (sc-33673) at 1:3000, Synaptosomal-associated protein, 25 kDa (SNAP25) (sc-20038) at 1:3000, synaptophysin (SYP) (sc-17750) at 1:3000, postsynaptic density protein-95 (PSD-95) (sc-71933) at 1:3000, p-NF-kB (sc-136548) at 1:1000, TLR4 (sc-293072) at 1:1000, TNFα (sc-52746) at 1:1000 (Santa Cruz, Dallas, TX, USA), iNOS (610432) at 1:3000 (BD Lifescience, Franklin Lakes, NJ, USA), COX-2 (160126) at 1:3000 (Cayman, Ann Arbor, MI, USA).

### 2.10. RNA Isolation and Quantitative PCR

After extracting total RNA from microglial cell line BV2 with TRIzol reagent (Invitrogen, Carlsbad, CA, USA), complementary DNA was synthesized from 2 μg of total RNA. Quantitative polymerase chain reaction (qPCR) was performed with SYBR Green I (GeNet Bio, Nonsan, Korea) in a LightCycler 480 system (Roche, Rotkreuz, Switzerland). Relative expression of mRNA was measured using comparative CT (ΔΔCT), normalized to GAPDH, and described as the fold change over control. Primers are as follows [28]; *GAPDH*, Forward (5′-3′) GTG-GCA-AAG-TGG-AGA-TTG-TTG, Reverse (5′-3′) CGT-TGA-ATT-TGC-CGT-GAG-TG; *TNFα*, Forward (5′-3′) TGG-GAC-AGT-GAC-CTG-GAC-TGT, Reverse (5′-3′) TTC-GGA-AAG-CCC-ATT-TGA-GT; *IL-1β*, Forward (5′-3′) AAG-GGC-TGC-TTC-CAA-ACC-TTT-GAC, Reverse (5′-3′) ATA-CTG-CCT-GCC-TGA-AGC-TCT-TGT; *IL-6*, Forward (5′-3′) ATC-CAG-TTG-CCT-TCT-TGG-GAC-TGA, Reverse (5′-3′) TAA-GCC-TCC-GAC-TTG-TGA-AGT-GGT; *iNOS*, Forward (5′-3′) GCT-CAT-GCG-GCC-TCC-TTT, Reverse (5′-3′) CCT-GGT-ACG-GGC-ATT-GCT; *COX-2*, Forward (5′-3′) TGC-TGT-ACA-AGC-AGT-GGC-AA, Reverse (5′-3′) AGG-GCT-TTC-AAT-TCT-GCA-GCC-A.

### 2.11. Statistical Analysis

All data are presented as mean ± standard error of the mean (S.E.M) of the four independent experiments. Statistical significance was calculated by a one-way ANOVA with Tukey’s post hoc test for comparison between more than two conditions, where applicable. All statistical analysis was done using GraphPad Prism 8.0 software (GraphPad, San diego, CA, USA). *p*-value < 0.05 was considered statistically significant. 

## 3. Results

### 3.1. Pharmacological Inhibition of Syk Mitigated the Expression of Microglial Cells and Inflammatory Mediators in LPS-Induced Mouse

**Figure 2:**
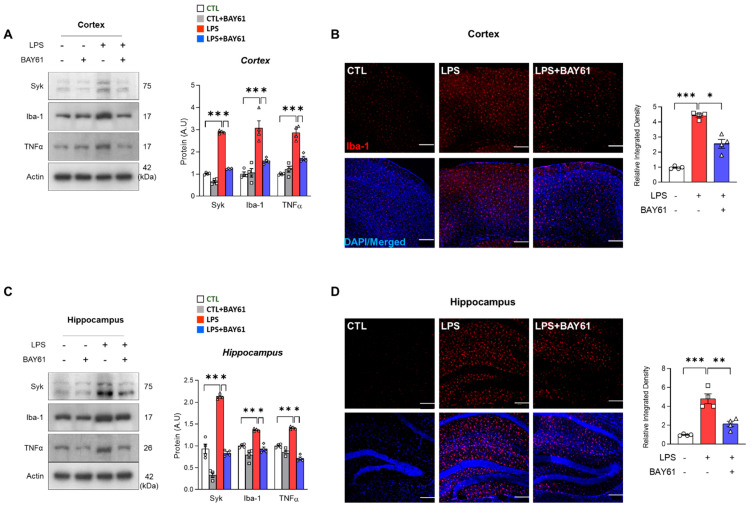
Effects of Syk against LPS-induced neuroinflammation in mice models. Western blot results of of Syk, Iba-1, and TNFα in the mice (**A**) cortex and (**C**) hippocampus (*n* = 4 mice/per group). Representative immunofluorescence images of Iba-1 expression in the (**B**) cortex and (**D**) hippocampus of the experimental mice’s brains. Bar graph color, white: control, grey: control with BAY61-3606, red: LPS, blue: LPS with BAY61-3606. Scale bars, 100 μm. Values indicate the mean ± SEM. The significant differences have been given in the graphs as follows: * *p* < 0.05, ** *p* < 0.005, *** *p* < 0.0001 compared with a group of interests and determined by one-way ANOVA followed by Tukey’s multiple comparison test. Abbreviation: Syk, tyrosine-protein kinase; LPS, lipopolysaccharide; Iba-1, ionized calcium-binding adaptor molecule 1; TNFα, tumor necrosis factor alpha.

In the brain microglial cells, Syk was highly expressed in response to ischemic stroke or LPS [16,29]. Consistent with previous findings, Syk was increased in conjunction with microglia activation marker Iba-1 and pro-inflammatory cytokine TNFα in the cortex and the hippocampus (Figure 2A,C; Supplementary Figure S1A,B). Next, we checked whether LPS-induced microglia activation was suppressed via inhibition of Syk. Our data showed that inhibition of Syk (via BAY61-3606) mitigated the expression of Syk as well as Iba-1 and TNFα, indicating that Syk inhibition reduces microglia-mediated neuroinflammation in LPS-induced mice cortex and hippocampus (Figure 2A,C; Figure S1A,B). In the immunofluorescence experiment, Iba-1 positive area of cortex and hippocampus were significantly decreased by Syk inhibition compared to the LPS alone-treated mice cortex and hippocampus (Figure 2B,D). 

### 3.2. Pharmacological Inhibition of Syk Mitigated the Inflammatory Mediators in LPS-Induced Mouse

**Figure 3:**
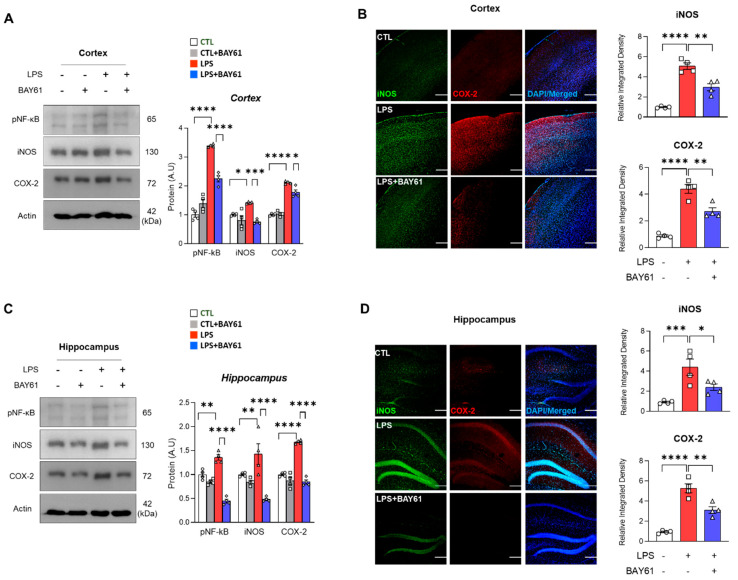
Pharmacological inhibition of Syk mitigates LPS-induced inflammatory mediators in the mice brain. Representative images of (**A**) immunoblot and (**B**) immunofluorescence of inflammatory mediators (p-NF-κB, iNOS, COX-2) in LPS-treated model with or without BAY61-3606 (*n* = 4) in cortex. Representative images of (**C**) immunoblots (**D**) and immunofluorescence of the inflammatory mediators in LPS-treated model with or without BAY61-3606 (*n* = 4) in hippocampus. Bar graph color, white: control, grey: control with BAY61-3606, red: LPS, blue: LPS with BAY61-3606. Scale bars, 100 μm. Values indicate the mean ± SEM. The significant differences have been given in the graphs as follows: * *p* < 0.05, ** *p* < 0.005, *** *p* < 0.0001, **** *p* < 0.00001 compared with a group of interests and determined by one-way ANOVA followed by Tukey’s multiple comparison test. Abbreviations: Syk, tyrosine-protein kinase; LPS, lipopolysaccharide; p-NF-κB, phosphor-nuclear factor kappa B; iNOS, inducible nitric oxide synthase; COX-2, cyclooxygenase-2.

As shown in Figure 2, that inhibition of Syk was responsible for the suppression of microglial cells activation. Next, we investigated whether inflammatory mediators are regulated by Syk inhibition. As shown in Figure 3, LPS-induced phosphorylation of NF-κB was ameliorated by Syk inhibition, followed by downregulation of iNOS and COX-2 in LPS-induced cortex and hippocampus (Figure 3A–D).

### 3.3. Pharmacological Inhibition of Syk Mitigated LPS-Induced Inflammation in BV2 Cells

**Figure 4:**
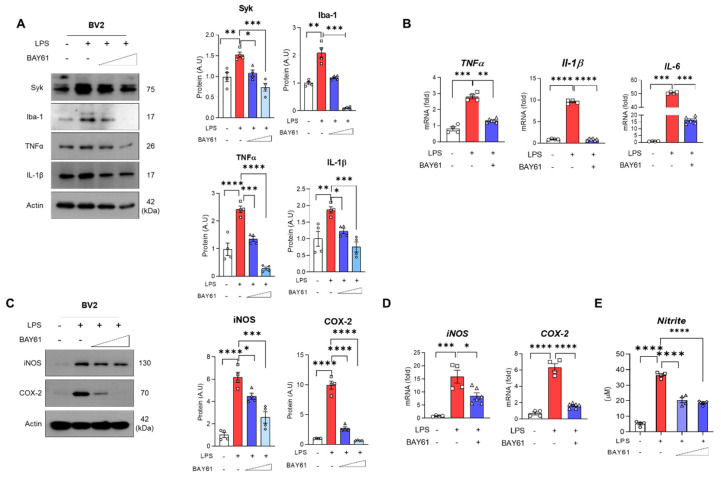
Pharmacological inhibition of Syk mitigates LPS-induced inflammation in BV2 microglial cells. (**A**) Western blot results of BV2 cells exposed to LPS (100 ng/mL) and BAY61-3606 (10 nM or 100 nM) for 24 h with respective graphs of Syk, Iba-1, TNFα, and IL-1β (*n* = 4). (**B**) Quantitative analysis of mRNA expressions of inflammatory cytokines; TNFα, Il-1β, and IL-6 (CTL, *n* = 4; LPS, *n* = 4; LPS+BAY61-3606, *n* = 6). After treating LPS (100 ng/mL) and BAY61-3606 (10 nM or 100 nM) for 24 h, Media and cell lysates were separately collected. (**C**) Immunoblot and quantification of iNOS and COX-2 (**D**) mRNA of iNOS and COX-2 were measured by qPCR after treating LPS (100 ng/mL) and BAY61-3606 (100 nM) for 6 h (CTL, *n* = 4; LPS, *n* = 4; LPS+BAY61-3606, *n* = 6). (**E**) Nitrite concentration was determined in the media using Griess reagent. (*n* = 4). Bar graph color, white: control, grey: control with BAY61-3606, red: LPS, blue: LPS with BAY61-3606. Values indicate the mean ± SEM. The significant differences have been given in the graphs as follows: * *p* < 0.05, ** *p* < 0.005, *** *p* < 0.0001, **** *p* < 0.00001 compared with a group of interests and determined by one-way ANOVA followed by Tukey’s multiple comparison test. Abbreviations: Syk, tyrosine-protein kinase; LPS, lipopolysaccharide; Iba-1, ionized calcium-binding adaptor molecule 1; TNFα, tumor necrosis factor alpha; IL, interleukin; iNOS, inducible nitric oxide synthase; COX-2, cyclooxygenase-2.

Next, we investigated whether BAY61-3606 suppresses LPS-induced inflammation in BV2 cells. LPS-induced Syk is down regulated by BAY61-3606 in a dose-dependent manner (Figure 4A). Likewise, Iba-1 and TNFα, and IL-1β expression in the BV2 cells were decreased by BAY61-3606 in a dose-dependent manner (Figure 4A). We also checked the mRNA expressions of pro-inflammatory cytokines, which showed that the expression of TNFα, Il-1β, and Il-6 were reduced with inhibition of Syk (Figure 4B). To further investigate the effects of Syk inhibition on the pro-inflammatory cytokines, we conducted a cytokine array using BV2 cell lysates. According to our findings, LPS increased the expression of interferon gamma-induced protein 10 (IP-10), monocyte chemoattractant protein-1 (MCP-1), chemokine CC motif ligand 3 (CCL3), CCL4, CXC motif chemokine 2 (CXCL2), but were significantly reduced with the inhibition of Syk (Figure S2A). Furthermore, LPS stimulated iNOS protein and mRNA expression followed by elevated levels of nitrite, as well as COX-2 protein and mRNA levels. When the Syk inhibitor was treated, both protein and mRNA levels of iNOS and COX-2 were significantly decreased (Figure 4C,D) and nitrite was significantly suppressed as well (Figure 4E). Collectively, Syk is a central inflammatory regulator in microglia.

### 3.4. Pharmacological Inhibition of Syk Mitigated the Apoptotic Cell Death in LPS-Induced Mice

**Figure 5:**
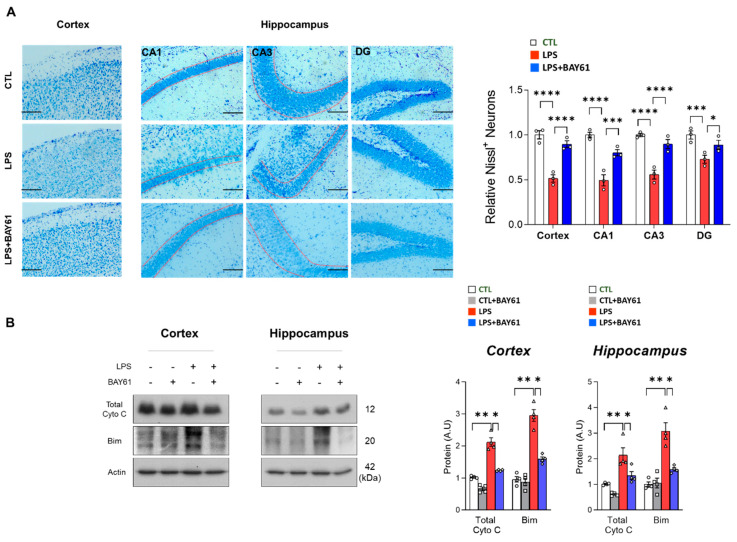
BAY61-3606 alleviates LPS-induced neuronal cell death. (**A**) Representative Nissl images of cortex and specific hippocampal regions (Cornu ammonis (CA)1, CA3, and dentate gyrus (DG)) after intraperitoneal injection of LPS (0.25 mg/kg per animal) and BAY61-3606 (10 mg/kg) for 9 days (*n* = 3). Scale bars, 50 μm. (**B**) Immunoblot of cytochrome C and Bim in the cortex and hippocampus. Bar graph color, white: control, grey: control with BAY61-3606, red: LPS, blue: LPS with BAY61-3606. Values indicate the mean ± SEM. The significant differences have been given in the graphs as follows: * *p* < 0.05, ** *p* < 0.005, *** *p* < 0.0001, **** *p* < 0.00001 compared with a group of interests and determined by one-way ANOVA followed by Tukey’s multiple comparison test. Abbreviations: Syk, tyrosine-protein kinase; LPS, lipopolysaccharide; Bim, BCL-2-like protein 11.

Next, we investigated whether inhibition of Syk ameliorates LPS-induced neuronal cell death. According to the Nissl staining, LPS markedly reduced neuronal cell density in the cortex and hippocampus compared to the control group (CA1 and CA3 regions). However, dentate gyrus (DG) observed no differences. Nevertheless, the reduced neuronal density was recovered with the inhibition of Syk through BAY61-3606, compared to the LPS-induced mice (Figure 5A). Consistent with histological analysis, apoptotic-associated protein cytochrome C (Cyto C) and Bcl-2-like protein 11 (Bim) were significantly downregulated by Syk inhibition in LPS-induced cortex and hippocampus (Figure 5B). Additionally, inhibition of Syk suppressed caspase 3, involved in the execution of apoptosis, in LPS-induced mouse cortex and hippocampus (Figure S3A,B).

### 3.5. Pharmacological Inhibition of Syk Protected against Neurotoxin-Mediated Neuronal Cell Death

**Figure 6:**
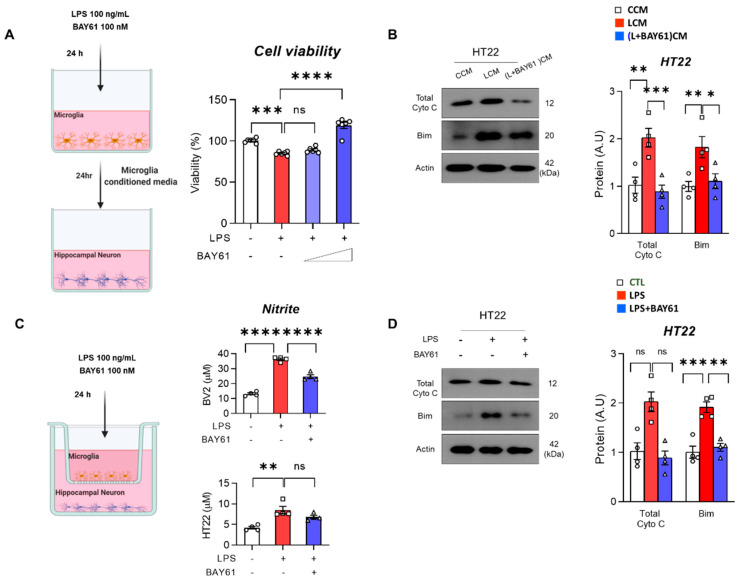
BAY61-3606 alleviates neurotoxin-mediated neurodegeneration in BV2. (**A**) Experimental scheme for microglia conditioned medium. Briefly, LPS (100 ng/mL) and BAY61-3606 (10 nM or 100 nM) were treated in BV2 for 24 h. After collecting microglia conditioned medium, it was treated by replacing original medium of hippocampal cell line HT22 for 24 h (left), and cell viability (%) was measured using MTT assay (right). (**B**) Immunoblot of Total Cyto C and Bim to investigate neuronal cell death in HT22 cells exposed to LPS-treated microglia conditioned medium (LCM) (*n* = 4). (**C**) Experimental schemes of transwell-based co-culture system of BV2 and HT22 (left). Nitrite was measured at transwell permeable supports containing BV2 and plate containing HT22 (right). (**D**) Immunoblot of Total Cyto C and Bim to detect neuronal cell death by paracrine manner of LPS-induced microglia activation. Bar graph color, white: control, red: LPS, blue: LPS with BAY61-3606. Values indicate the mean ± SEM. The significant differences have been given in the graphs as follows: ns: not significant, ns, non-significant, * *p* < 0.05, ** *p* < 0.005, *** *p* < 0.0001, **** *p* < 0.00001 compared with a group of interests and determined by one-way ANOVA followed by Tukey’s multiple comparison test. Abbreviations: Syk, tyrosine-protein kinase; LPS, lipopolysaccharide; Bim, BCL-2-like protein 11.

Based on our previous results, we hypothesized that suppression of neuronal cell death may be dependent upon microglia activation by a paracrine manner. We introduced two strategies: conditioned medium and transwell culture. We treated LPS in BV2 with or without BAY61-3606. After 24 h, the conditioned medium was collected and treated in mouse hippocampal HT22 cells (Figure 6A left). LPS-conditioned media significantly promoted neuronal cell death, which was significantly reversed only in the high dose inhibitor of Syk compared to the LPS-conditioned media. Nevertheless, the findings indicate that neurotoxin derived from LPS-induced microglia was decreased by BAY61-3606 (Figure 6A right). Subsequently, we collected HT22 cells, exposed them to desired conditioned media, and investigated apoptotic-associated proteins Cyto C and Bim. We found that LPS-conditioned media (LCM) dramatically promoted apoptotic cell death, which was significantly reversed with the administration of BAY61-3606, thus supporting cell viability data (Figure 6B). Next, BV2 cells were seeded in transwell permeable supports and stimulated with LPS and BAY61-3606. Then, transferred supports were placed into a plate containing mouse hippocampal HT22 (Figure 6C left). BV2 nitrite concentration supported with aforementioned findings. However, reduction of nitrite concentration did not show in the well containing HT22 (Figure 6C right). LPS stimulation in the permeable supports did promote increase of Total Cyto C and Bim, and BAY61-3606 reversed these protein expressions (Figure 6D).

### 3.6. Pharmacological Inhibition of Syk Restored Synaptic Dysfunctions, Cognitive, and Spatial Working Memory Impairment in LPS-Injected Mice

**Figure 7:**
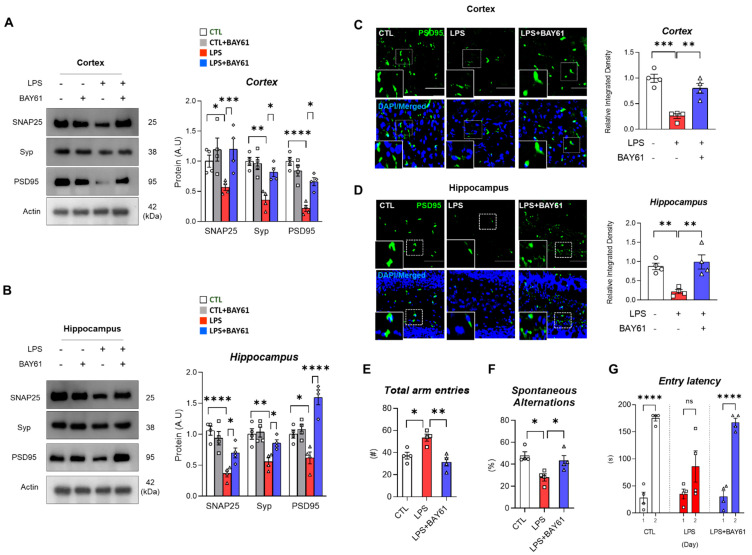
BAY61-3606 improves LPS-induced synaptic dysfunction and memory impairments. Immunoblot and quantification of SNAP25, Syp and PSD95 in LPS-treated model with or without BAY61-3606 in (**A**) cortex (*n* = 4) and (**B**) hippocampus (*n* = 4). Representative immunofluorescence images of (**C**) cortex and (**D**) hippocampus treated with PSD95 antibodies. Scale bars, 25 μm. Spatial working memory was measured by Y-maze test. In the test, (**E**) total arm entries, (**F**) spontaneous alternations were measured by automatic video tracking system. (**G**) Learning memory was measured by fear-aggravated test: passive avoidance. Bar graph color, white: control, grey: control with BAY61-3606, red: LPS, blue: LPS with BAY61-3606. Values indicate the mean ± SEM. The significant differences have been given in the graphs as follows: ns: not significant, * *p* < 0.05, ** *p* < 0.005, *** *p* < 0.0001, **** *p* < 0.00001 compared with a group of interests and determined by one-way ANOVA followed by Tukey’s multiple comparison test. Abbreviations: LPS, lipopolysaccharide; SNAP25, Synaptosomal-associated protein, 25 kDa; Syp, synaptophysin; PSD-95, postsynaptic density protein-95.

Memory impairments are highly associated with synaptic dysfunction followed by neuronal cell death [30]. Since we found that Syk inhibition suppressed LPS-induced neuroinflammation and neuronal cell death, next we checked whether inhibition of Syk has an impact on the synaptic and cognitive dysfunction in LPS-induced mice. As shown in Figure 7, there was a significant reduction in the expression of synaptosome-associated protein, 25 kDa (SNAP25), synaptophysin (Syp), and postsynaptic density protein-95 (PSD95) in the LPS-injected mice brains compared to the saline-injected control group (Figure 7 A–D). Interestingly, these markers were significantly restored after administration of BAY61-3606 (Figure 7A,B). Immunofluorescence data showed that reduced PSD95 was significantly recovered by BAY61-3606 treatment in LPS-treated mice cortex and hippocampus (Figure 7C,D). To check the effects of Syk inhibition on memory-related functions in LPS-induced mice, we performed two kinds of behavioral tests: Y-maze test and passive avoidance test. In the Y-maze, we investigated the spontaneous alternation related to short-term spatial working memory, and the passive avoidance test associated with learning behaviors. According to our findings, the total number of arm entries were significantly increased (Figure 7E), and the percentage of alternation and exploration were significantly decreased in LPS-induced mice compared to the saline-injected control group (Figure 7F). Mobility and total distance in Y-maze zone have not shown differences between mice groups (Figure S4A,B). As expected, LPS-induced with BAY61-3606 mice exhibited restoration of spatial working memory impairments compared to LPS-induced mice (Figure 7E,F; Figure S4C). In a passive avoidance test, LPS-induced mice did not stay in the light compartment, although the mice were exposed to electric shock on the previous day. Syk inhibition promotes time to stay light compartment, which is comparable to control mice (Figure 7G). 

## 4. Discussion

The aim of the current study was to investigate the effects of Syk inhibition, through pharmacological Syk inhibitor BAY61-3606, against the LPS-induced neuroinflammation and neurodegeneration in both in vivo and in vitro models. For the induction of microglia activation, we used LPS, which is a known neurotoxin and has been used in a wide range of pre-clinical studies [31,32]. The present study demonstrated that LPS promotes Syk expression in the cortex and hippocampus and in the BV2 microglial cell line. LPS-induced microglia activation is regulated by Syk, and pharmacological inhibition of Syk mitigated both inflammatory mediators and pro-inflammatory cytokines. Moreover, inhibition of Syk protected against apoptotic cell death, synaptic dysfunction, and cognitive impairment. 

Neuroinflammation is one of the typical symptoms of neurodegenerative diseases such as AD and PD. To reflect neurodegenerative disease model using LPS, most studies have introduced high dose of LPS with a single injection. Though high dose of LPS can dramatically induce neuroinflammation, there is a risk of septic shock [33,34]. Therefore, high dose of LPS is insufficient to reflect neurodegenerative features such as cognitive dysfunction. We consistently continued to study the neurodegenerative phenotype in a model treated with low concentrations of LPS [31,35,36,37]. In this study, we revealed how neuroinflammation is caused by changes in Syk expression and is related to neurodegenerative features using the established LPS-induced neurodegeneration model.

Since microglia activation exhibits neurotoxicity and has a direct impact on mitochondrial quality control and energy homeostasis [38], it promotes cell death of adjacent neuron. Therefore, we investigated the neuronal density and its expression of apoptotic markers in the LPS-induced models. The execution of mitochondrial apoptosis with the administration of LPS is consistent with the previous study [39]. Our results demonstrated that BAY61-3606 restored LPS-induced neuronal cell death through regulating cytochrome C and Bim in the LPS-injected mouse cortex and hippocampus. Based on the data that microglia-mediated inflammatory mediators were markedly mitigated by Syk inhibition, neuronal cell survival might be increased due to inhibition of neurotoxic effect by inflammatory mediators. As expected, present data showed that neuronal cell survival is dependent upon microglia activation. Additionally, activated microglia induced the secretion of toxic inflammatory mediators including NO from iNOS and PGE2 from COX-2, which are responsible for neuronal cell death and are target genes of NF-κB [40]. This aligns with our data, since we showed that inflammatory mediators are regulated by Syk dependent manner, consistent with other Syk inhibitors from other pathological conditions [16,29].

Another function of microglia is phagocytosis. Phagocytic activity has been considered as a beneficial effect to maintain brain homeostasis. In fact, aggregation of dead or dying neurons or abnormal proteins are removed by microglia. Dysregulation of phagocytosis causes neuroinflammation and neurodegeneration since those debris constantly stimulate microglia to be activated towards M1 microglia status [41]. The M1 microglia has characteristics of secreting pro-inflammatory cytokines, including TNFα, IL-6, and IL-1β, as well as impaired phagocytic activity [42]. Our data supported the M1 characteristics because our data showed an increase of Iba-1 and TNFα in the cortex and hippocampus, as well as an increase in Iba-1, TNFα, IL-1β, and IL-6 in the BV2 cell line. Although more research is needed, in general, phagocytosis increases when microglia are in M2 polarization [43].

Lastly, multiple studies have shown that LPS inhibits synaptic protein and causes cognitive impairment in vivo [8,40,41]. As neuronal cell death progresses, the synaptic function of the neuron is lowered, and accordingly, cognitive and memory impairments occur. In the present study, it was confirmed that the synaptic proteins of the cerebral cortex and hippocampus of LPS-induced mice were decreased. Since Syk-inhibited microglia reduced neuronal cell death through inhibition of inflammatory cytokines and inflammatory mediators, it was estimated that the expression of synaptic proteins would be restored. As expected, it was confirmed that the expression of presynaptic (Syp and SNAP25) and postsynaptic proteins (PSD95) was higher than in the LPS group. According to the expression pattern of synaptic proteins, LPS-induced cognitive and memory impairments improved by BAY61-3606 treatment after conducting mouse behaviors tests; Y-maze and passive avoidance test. 

Although no clinical trials have been conducted using BAY61-3606, several oral Syk inhibitors such as fostamatinib (R788), entospletinib (GS-9973), cerdulatinib (PTR062070), and TAK-659 are being tested for clinical trials [44]. The dose-limiting toxicity (DLT) were diarrhea, neutropenia, thrombocytopenia, increased lipase levels, generalized edema, mucositis, and increased aspartate aminotransferase (AST) [45]. Since BAY61-3606 has shown protective properties, future studies could investigate its effect in clinical trials.

## 5. Conclusions

Collectively, the findings clearly show the potential role of Syk in the pathophysiology of neuroinflammation and neurodegeneration, and highlight the therapeutic potentials of BAY61-3606 in the regulation of neuroinflammatory and neurodegenerative diseases, including AD and PD.

## Data Availability

The authors hereby declares that the data presented in this study will be presented upon request from the corresponding author.

## Supplementary Materials

The following supporting information can be downloaded at: https://www.mdpi.com/article/10.3390/cells11111777/s1: Figure S1: Syk is associated with inflammatory cytokines and mediators; Figure S2: BAY61-3606 suppressed LPS-induced pro-inflammatory cytokines; Figure S3: LPS-induced Caspase 3 is suppressed by BAY61-3606; Figure S4: Effects of BAY61-3606 on LPS-induced spatial learning and memory impairments.
