# Supplementary material for: Pharmacological Inhibition of Spleen Tyrosine Kinase Suppressed Neuroinflammation and Cognitive Dysfunction in LPS-Induced Neurodegeneration Model
Source: Cells. 2022 May 28;11(11):1777. doi: 10.3390/cells11111777 (PMC9179326; doi:10.3390/cells11111777)
Supplement: Supplementary file 1 [file cells-11-01777-s001.zip › cells-1709593-supplementary.pdf]

cells

MDPI

Supplementary Files

Pharmacological Inhibition of Spleen Tyrosine Kinase Suppressed Neuroinflammation and Cognitive Dysfunction in LPS-induced Neurodegeneration Model

Min Woo Kim [1,†], Kyonghwan Choe [1,2,†], Jun Sung Park [1,†], Hyeon Jin Lee [1], Min Hwa Kang [1], Riaz Ahmad [1] and Myeong Ok Kim [1,3,*]

[1] Division of Life Sciences and Applied Life Science (BK 21 Four), College of Natural Science, Gyeongsang National University, Jinju 52828, Korea; mwkim0322@gnu.ac.kr (M.W.K.); k.choe@maastrichtuniversity.nl (K.C.); jsp@gnu.ac.kr (J.S.P.); lhj4912@gnu.ac.kr (H.J.L.); kmh1020@gnu.ac.kr (M.H.K); riazk0499@gnu.ac.kr (R.A.)
[2] Department of Psychiatry and Neuropsychology, School for Mental Health and Neuroscience (MHeNs), Maastricht University, Maastricht 6229ER, The Netherlands
[3] Alz-Dementia Korea Co., Jinju 52828, Korea
* Correspondence: mokim@gnu.ac.kr; Tel.: +82-55-772-1345
† These authors contributed equally to this paper.

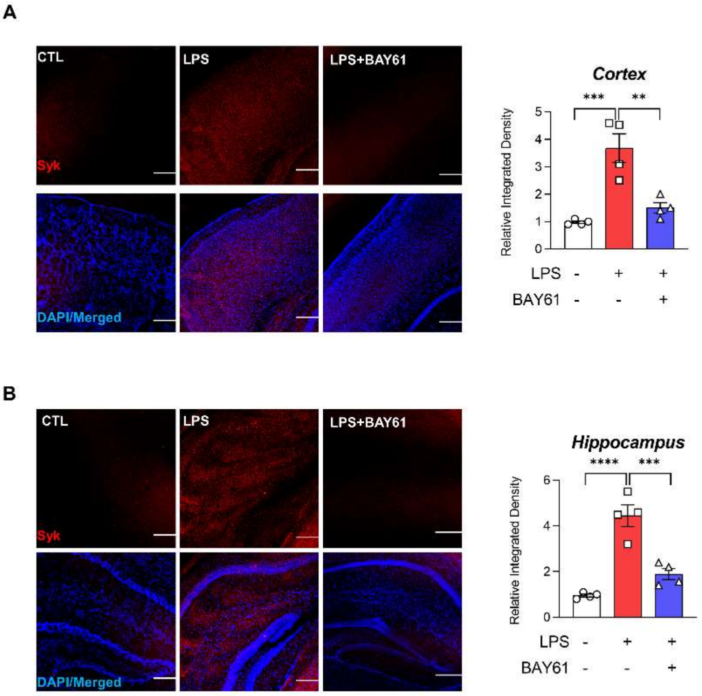

Figure S1. LPS-induced Syk expression is suppressed by BAY61-3606. Representative immunofluorescence images of Syk expression in (**A**) cortex and (**B**) hippocampus in LPS-induced mice with or without BAY61-3606. Scale bars, 100 µm. Values indicate the mean ± SEM, * $p < 0.05$, ** $p < 0.005$, *** $p < 0.0001$, **** $p < 0.00001$ compared with a group of interests and determined by one-way ANOVA followed by Tukey's multiple comparison test.

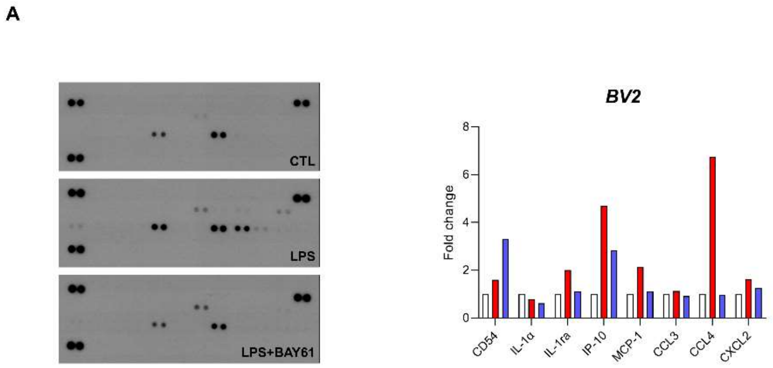

Figure S2. BAY61-3606 suppressed LPS-induced pro-inflammatory cytokines. (**A**) Dot blot of proteomic profiles for detecting inflammatory cytokines and quantification (Average of two independent experiments).

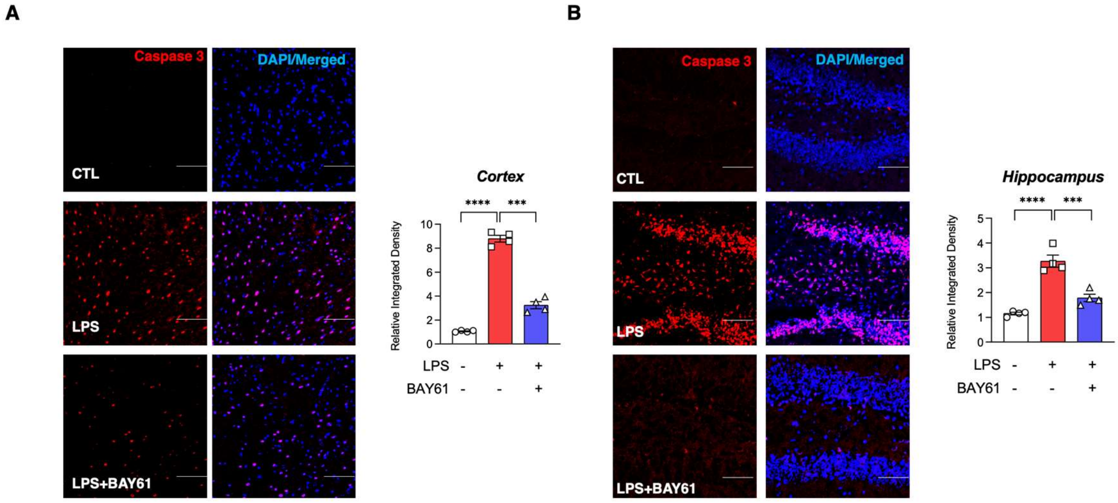

Figure S3. LPS-induced Caspase 3 is suppressed by BAY61-3606. Representative immunofluorescence images of Caspase 3 expression in (**A**) cortex and (**B**) hippocampus in LPS-induced mice with or without BAY61-3606. Scale bars, 25 μm Values indicate the mean ± SEM, *** $p < 0.0001$, **** $p < 0.00001$ compared with a group of interests and determined by one-way ANOVA followed by Tukey's multiple comparison test.

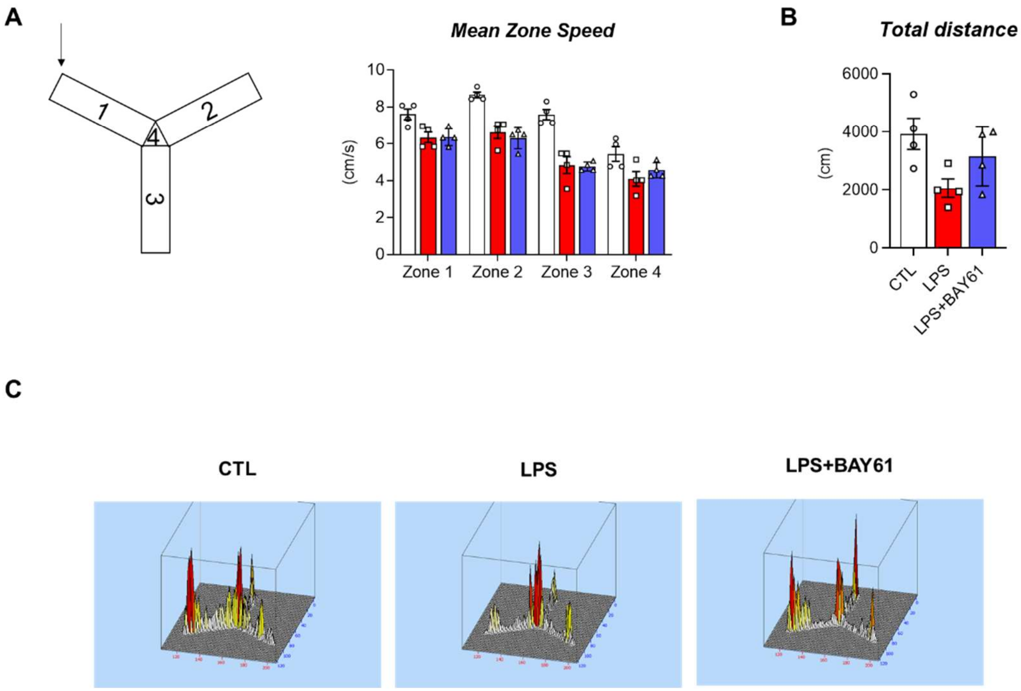

Figure S4. Effects of BAY61-3606 on LPS-induced spatial learning and memory impairments. (**A**) Definition of Y-maze zone (left) and mice speed in each zone. (**B**) Total distance in Y-maze zone. (**C**) Trajactory map of mice movements in Y-maze.
